# Temporally Unpredictable Sounds Exert a Context-Dependent Influence on Evaluation of Unrelated Images

**DOI:** 10.1371/journal.pone.0131065

**Published:** 2015-06-22

**Authors:** Dominik R. Bach, Erich Seifritz, Raymond J. Dolan

**Affiliations:** 1 Wellcome Trust Centre for Neuroimaging, University College London, London, United Kingdom; 2 University Hospital of Psychiatry, University of Bern, Bern, Switzerland; 3 Department of Psychology and Ergonomics, Berlin Institute of Technology, Berlin, Germany; 4 Department of Psychiatry, Psychotherapy, and Psychosomatics, University of Zurich, Zurich, Switzerland; Durham University, UNITED KINGDOM

## Abstract

Temporally unpredictable stimuli influence murine and human behaviour, as previously demonstrated for sequences of simple sounds with regular or irregular onset. It is unknown whether this influence is mediated by an evaluation of the unpredictable sound sequences themselves, or by an interaction with task context. Here, we find that humans evaluate unrelated neutral pictures as more negative when these are presented together with a temporally unpredictable sound sequence, compared to a predictable sequence. The same is observed for evaluation of neutral, angry and fearful face photographs. Control experiments suggest this effect is specific to interspersed presentation of negative and neutral visual stimuli. Unpredictable sounds presented on their own were evaluated as more activating, but not more aversive, and were preferred over predictable sounds. When presented alone, these sound sequences also did not elicit tonic autonomic arousal or negative mood change. We discuss how these findings might account for previous data on the effects of unpredictable sounds, in humans and rodents.

## Introduction

Forecasting the state of the world is fundamental for all organisms. Because environments differ in the degree to which events are predictable, humans and other animals can maximise predictability by, for example, moving from one environment to another. A large literature shows that organisms actively prefer predictable over unpredictable salient events such as punishments and rewards [1, 2]. Such observations fit a class of theories which invoke reduction of uncertainty [3] as a prime motivating force in a wide class of behaviours [4, 5].

Much experimental evidence on uncertainty avoidance relies on the use of unpredictable salient events such that unpredictability has rarely been assessed per se. In fact, it has been argued that a preference for predictable salient events does not reflect a propensity to reduce uncertainty, but instead a propensity to maximise periods of safety. Such safety is lacking from environments characterised by unpredictable salient events [6]. This explains why animals only avoid an unpredictable environment when rewards are infrequent [7]. In contrast, when rewards are very frequent and hence periods of non-reward are short, other factors may dominate and animals can even prefer an unpredictable environment [7]. Within this perspective, neutral events that are not motivationally salient do not engender lack of safety, so their unpredictability has no consequence. Also humans may prefer unpredictability because complete predictability is perceived as monotonous and boring [8, 9]. Psychophysical findings, on the other hand, indicate that perceptual noise, which by definition engenders unpredictability, improves visual perception [10–14]. Hence from a normative perspective, it can be beneficial for an organism to seek a degree of environmental unpredictability rather than its complete absence.

We previously investigated responses to unpredictability per se by exposing mice and humans to a stream of temporally predictable (i. e. regular onsets), or unpredictable (i. e. random onsets), simple beep sounds [15], where each single beep sound in itself is an affectively neutral event. Mice actively avoided the unpredictable sequence, and exhibited increased anxiety-like behaviour in the elevated plus maze [15]. Humans, on the other hand, showed increased attentional bias towards threat in a dot probe task when hearing such a sequence [15], a phenomenon also seen in states of anxiety [16, 17]. These observations were taken to suggest that sound sequences were aversive and anxiogenic in mice and humans. However, an alternative interpretation of the human findings is that unpredictable sounds changed the evaluation of the threatening stimulus in the dot probe task. This altered evaluation could explain previous findings without necessarily invoking anxiety. Here, we revisit this question by directly assessing evaluation of negative and neutral stimuli during presentation of temporally predictable and unpredictable sounds. In particular, by asking participants to explicitly evaluate images while hearing the sound sequences, we sought to address whether, and by which mechanism, valence appraisal is altered. To answer whether the sound sequences are anxiogenic or aversive by themselves, we examine, in control experiments, the following: behavioural tendencies, subjective feeling and tonic sympathetic arousal, associated with the sound sequences themselves.

## General Methods

### Participants

Table 1 provides an overview of the 6 experiments. By advertising participation in a study that assesses the impact of noise in a reaction time task, we recruited, for each experiment, 20–25 participants per sound condition. Samples 5 and 6 overlapped, all other samples were completely independent. State and trait anxiety were controlled for with the state-trait anxiety inventory (STAI; [18]) in experiments 1, 4–6, and had no impact on influences of the sound sequences when taken into account as covariate. Skin conductance data from experiments 1–4, pooled across the sound conditions, was included into methodological investigations which we published separately [19, 20].

**Table 1:** Design overview of the 6 experiments.

**Experiment 1**	*Group 0*	*Group 1*:	*Group 2*
	Silence	Predictable	Unpredictable
	Tonic arousal: SF		
	Phasic arousal and stimulus ratings: Neutral/Negative pictures		
**Experiment 2**	*Group 0*	*Group 1*	*Group 2*
	Silence	Predictable	Unpredictable
	Tonic arousal: SF		
	Phasic arousal and stimulus ratings: Neutral pictures		
**Experiment 3**		*Group 1*	*Group 2*
		Predictable	Unpredictable
		Phasic arousal and stimulus ratings: Negative/Neutral/Positive pictures	
**Experiment 4**		*Group 1*	*Group 2*
		Predictable	Unpredictable
		Tonic arousal: SF	
		Phasic arousal and stimulus ratings: Neutral/Fearful/Angry face photographs	
**Experiment 5**	Forced choice: Predictable/Unpredictable		
	Post-hoc stimulus ratings		
**Experiment 6**	Silence/Predictable/Unpredictable (in balanced order)		
	Tonic arousal (SF, HR)/Mood changes		

### Ethics statement

All participants were adults who gave written informed consent. The study (including the form of consent) was approved by local research ethics committees (Kantonale Ethikkomission Bern, NHS Joint National Hospital for Neurology and Neurosurgery and UCL Institute of Neurology Ethics Committee, Kantonale Ethikkomission Zurich). All participants were debriefed to the aims of the study.

### Sound sequences

In all experiments, we used a predictable sequence of neutral beep sounds (1000 Hz, 40 ms duration, 5 ms ramps between on/offset and full volume) presented with an average repetition frequency of 5 Hz, i.e. an average stimulus onset asynchrony of 200 ms. For the unpredictable sequence, onset deviation was randomly drawn from a uniform distribution over ± 60 ms (experiment 1–4), ± 20 ms (experiment 5), or ± 40 ms (experiment 6). Sound sequences were produced using CSounds (www.csounds.com) and played over headphones with a loudness of ~80 dB (experiments 1–4) or ~85 dB (experiments 5–6). Experiments 5–6 were performed before experiments 1–4; in experiments 1–4 we increased onset jitter and slightly decreased loudness to maximise the experience of unpredictability, and minimise any possible aversiveness due to the beeps themselves. Experiments 1–4 realised a between-subjects design in which each participant heard one sound sequence for the entire duration of the experiment. In experiment 6, each participant heard all sound sequences in balanced order in a within-subjects design, while participants chose which sound sequence they heard in experiment 5.

### Skin conductance recording and analysis

We recorded skin conductance responses (SCR) on thenar/hypothenar surface of the non-dominant hand using Ag/AgCl cup electrodes and 0.5%-NaCl electrode paste [21, 22]. SCR data were analysed using PsPM/SCRalyze 2.1.8 (http://pspm.sourceforge.net) [23]. Tonic sympathetic arousal was estimated using dynamic causal modelling (DCM) for spontaneous fluctuations (SF) [24, 25], and was quantified as the number of spontaneous sudomotor impulses per epoch. Evoked phasic sympathetic arousal was estimated in a general linear convolution model [19, 26]. Event onsets were separately modelled as stick functions for each event type (neutral, aversive, break start, break end), convolved with a canonical skin conductance response function [27].

### Statistical analysis

Estimates of tonic arousal from experiments 1, 2, 4 and 6, as well as phasic sympathetic arousal and picture ratings from experiments 1–4, were analysed using ANOVAs (or ANCOVAs) in SPSS. We were interested in the difference between predictable and unpredictable stimuli such that this constituted our *a priori* contrast of interest for all experiments. In experiments 1–2, and 6, possible effects of mere auditory stimulation were analysed in an *a priori* contrast of predictable vs. silence. For analysis of phasic sympathetic arousal and picture ratings, experiments 1, 3–4 had an additional within-subjects factor *picture valence*. Experiment 5 was a forced-choice paradigm where we tested deviations from a uniform distribution of responses. We analysed time spent with unpredictable sound sequence, sound sequence that participants spent more time with (termed *preferred sequence*), and the ratings. Deviations from a uniform distribution were tested in SPSS with one-sample *t*-tests (continuous measures) and binomial tests (frequencies). In exploratory analyses, we examined the relation of state and trait anxiety on continuous response measures from experiment 5 in two separate simple regression models with anxiety as predictor. The impact on binary response measures was analysed with generalised linear models with anxiety as linear predictor, and logit link function–equivalent to logistic regression.

## Experiment 1

### Method

Experiment 1 followed a factorial design with the between-subjects factor sound type (silence/predictable/unpredictable) and the within-subject factor picture type (neutral, negative). We recruited 60 participants (30 male, 30 female; age: M = 23.7; SD = 4.7 years); one dataset was excluded due to technical malfunction. The experiment lasted about 20 minutes. During the first 2 minutes we obtained baseline skin conductance recordings for estimation of tonic arousal. Afterwards, participants watched, in randomised order, the 45 least arousing neutral (valence within 1 standard deviation around the mean) and 45 most arousing aversive pictures (valence lower than 1 standard deviation below the mean) from the International Affective Picture Set (IAPS, [28]) for 1 s each, with an inter stimulus (ISI) interval randomly determined as 7.65 s, 9 s, or 10.35 s. Participants were instructed to press the cursor up or down key on a computer keyboard to indicate whether they liked the picture or not. The experiment was divided into 3 blocks with 45 s breaks in between. Lists of images used in experiments 1–4 are available from the authors.

### Results & Discussion

**Fig 1:**
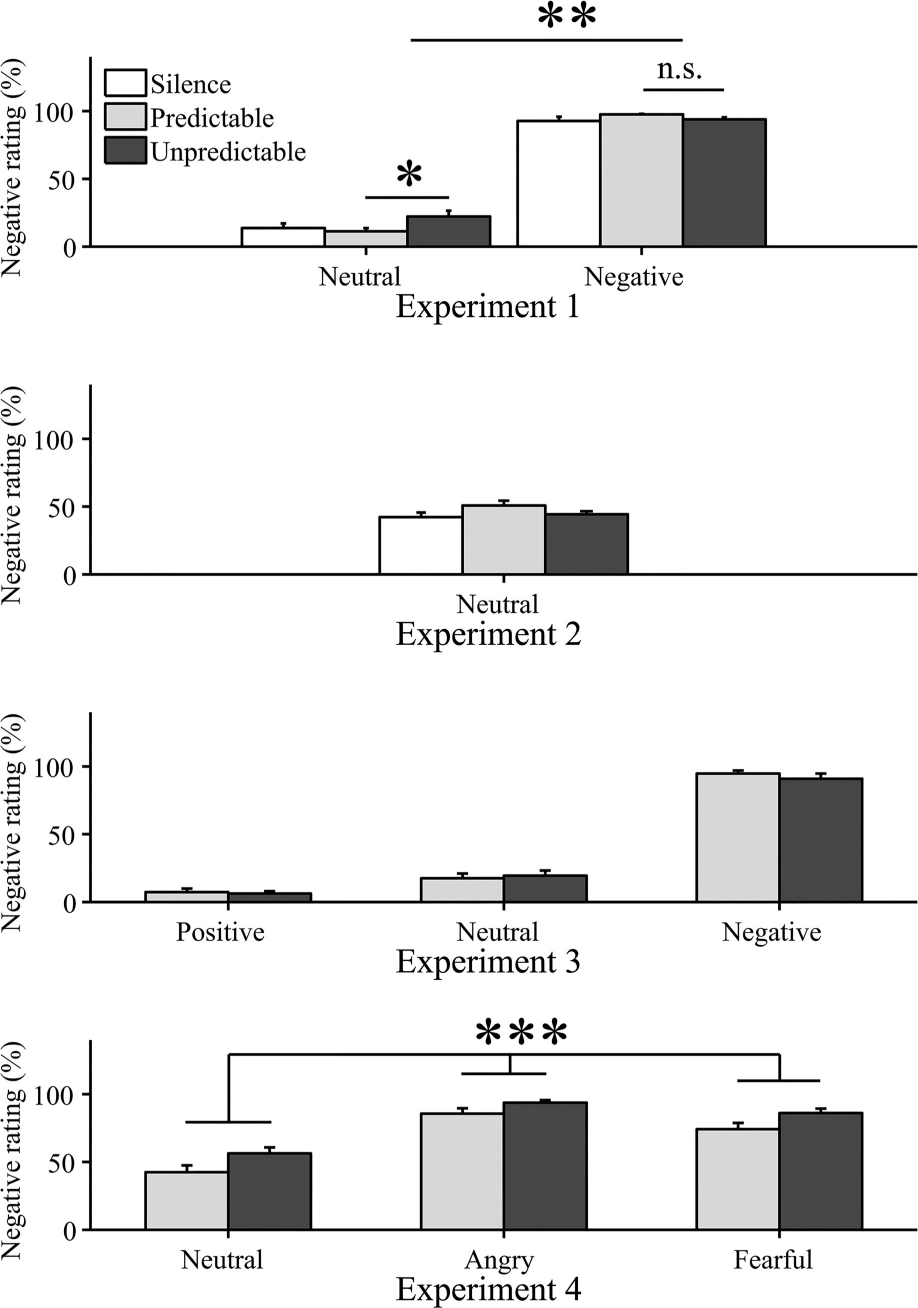
Stimulus ratings (mean ± standard error) for different pictures during silence, predictable, and unpredictable sound sequence. * p < .05, ** p < .01, *** p < .0005.

Across all conditions, aversive pictures were more often rated negative than neutral pictures (Tables 2–3, Fig 1). A valence × sound interaction meant that neutral pictures were more often rated negative in the unpredictable than in the predictable condition (post hoc t-test: t(37) = 2.6; p = .02, 2-tailed, uncorrected), with no difference for aversive pictures (post hoc t-test: t(37) = -1.8; p = .08, 2-tailed, uncorrected). This interaction survived (all p < .05) correction for baseline arousal, state anxiety, and trait anxiety in separate ANCOVAs that included both the covariate and its interaction with valence.

**Table 2:** Percentage of negative ratings, and reaction times (RT), and picture-associated phasic sympathetic arousal as estimated from skin conductance responses (SCR), for experiments 1–4.

			Silence			Predictable			Unpredictable		
Experiment	Measure	Stimulus	M	±	SD	M	±	SD	M	±	SD
Experiment 1	% negative	Neutral	15.6	±	15.8	12.9	±	11.4	25.8	±	19.4
		Negative	93.7	±	13.8	98.4	±	2.1	95.7	±	6.5
	RT (ms)	Neutral	983	±	230	989	±	285	1057	±	318
		Negative	871	±	249	943	±	339	963	±	319
	SCR (a.u.)	Neutral	0.022	±	0.133	0.009	±	0.106	0.288	±	0.476
		Negative	0.178	±	0.319	0.203	±	0.320	0.491	±	0.684
Experiment 2	% negative	Neutral	42.4	±	15.9	50.9	±	15.9	44.8	±	9.9
	RT (ms)	Neutral	1028	±	300	894	±	172	953	±	245
	SCR (a.u.)	Neutral	0.264	±	0.274	0.156	±	0.271	0.225	±	0.298
Experiment 3	% negative	Positive				7.6	±	11.5	6.6	±	7.6
		Neutral				18.4	±	15.1	18.7	±	17.3
		Negative				95.1	±	9.2	90.8	±	17.4
	RT (ms)	Positive				976	±	271	946	±	184
		Neutral				972	±	181	973	±	189
		Negative				890	±	281	881	±	148
	SCR (a.u.)	Positive				0.137	±	0.421	0.045	±	0.110
		Neutral				-0.025	±	0.404	-0.002	±	0.140
		Negative				0.353	±	0.395	0.140	±	0.141
Experiment 4	% negative	Neutral				16.1	±	8.9	21.4	±	7.5
		Angry				32.5	±	7.1	35.6	±	3.4
		Fearful				28.2	±	8.2	32.7	±	5.7
	RT (ms)	Neutral				1218	±	330	1040	±	295
		Angry				1121	±	358	899	±	282
		Fearful				1190	±	359	953	±	300
	SCR (a.u.)	Neutral				0.032	±	0.121	0.044	±	0.076
		Angry				0.003	±	0.087	0.027	±	0.049
		Fearful				0.036	±	0.094	0.048	±	0.069

**Table 3:** Experiment 1: Results from a 3 (sound sequence) × 2 (valence) repeated-measures ANOVA on stimulus ratings, reaction times (RT), and phasic sympathetic arousal as estimated from skin conductance responses (SCR); and from a one-way ANOVA on tonic sympathetic arousal as estimated from spontaneous fluctuations (SF) in skin conductance.

Rating	Valence	F (1,56) = 1101.8	η^2^ = .952	p < .001			
		unpredictable vs. predictable			predictable vs. silence		
	Sound	F (1,56) = 3.0	η^2^ = .051	p = .09	F (1,56) < 1	η^2^ = .002	n.s.
	Valence x Sound	F (1,56) = 7.4	η^2^ = .117	p < .01	F (1,56) = 1.7	η^2^ = .952	n.s.
RT	Valence	F (1,56) = 21.9	η^2^ = .282	p < .001			
		unpredictable vs. predictable			predictable vs. silence		
	Sound	F (1,56) = 1.7	η^2^ = .020	n. s.	F (1,56) = 2.3	η^2^ = .040	n.s.
	Valence x Sound	F (1,56) < 1	η^2^ = .004	n. s.	F (1,56) < 1	η^2^ = .003	n.s.
SCR	Valence	F (1,56) = 21.5	η2 = .277	p < .001			
		unpredictable vs. predictable			predictable vs. silence		
	Sound	F (1,56) = 6.0	η^2^ = .096	p < .05	F (1,56) < 1	η^2^ = .003	n.s.
	Valence x Sound	F (1,56) < 1	η^2^ = .000	n.s.	F (1,56) < 1	η^2^ = .000	n.s.
SF		unpredictable vs. predictable			predictable vs. silence		
	Sound	F (1,56) = 1.2	η^2^ = .022	n.s.	F (1,56) < 1	η^2^ = .008	n.s.

For the sound factor, only the a priori contrasts unpredictable vs. predictable and predictable vs. silence are tested.

Aversive pictures elicited stronger stimulus-evoked SCRs than neutral pictures across all conditions. SCRs to both aversive and neutral pictures were stronger in the unpredictable than in the predictable condition. This main effect survived correction (all p < .05) for baseline arousal, state anxiety, and trait anxiety. Tonic baseline arousal as indexed by SF, and reaction times (RT), were not influenced by the sounds. Thus there was no significant increase in tonic sympathetic arousal when the unpredictable sequence was presented alone. Phasic stimulus-associated sympathetic arousal was markedly increased in the unpredictable condition while stimulus ratings were more negative during the unpredictable sequence but only for neutral stimuli.

The combination of negative and neutral pictures was chosen in light of a previous investigation into unpredictable sound sequences [15]. Contradicting expectations, we found that only neutral picture ratings changed towards being reported more negative during the unpredictable sequence, while negative pictures were non-significantly rated less negative. This may suggest that more negative evaluation of images through the sound sequences is specific to neutral pictures. However, experiment 1 leaves open the possibility that the visual context of highly arousing pictures may have interacted with the impact of the sound sequence on neutral pictures, which was addressed in experiment 2.

## Experiment 2

We designed this experiment to replicate an alteration of neutral picture ratings found in experiment 1 while removing any impact of arousing visual context. Thus, we only assessed neutral pictures on their own, without negative stimuli interspersed.

### Method

Experiment 2 realised a one-way factorial design with the between-subjects factor sound type (silence/predictable/unpredictable). We recruited 61 participants (31 male, 30 female; age: M = 25.7; SD = 4.5 years). The experiment included only the 45 neutral pictures from experiment 1 with the same event timing and responses, and was divided into 3 blocks.

### Results & Discussion

Contrary to our expectations, we found that sound sequence had no impact on ratings, RT, tonic sympathetic arousal quantified as SF, or phasic stimulus-associated SCR. This suggests that the context of highly arousing IAPS pictures is relevant for the impact of the sound sequence on neutral picture appraisal in experiment 1, and also for the impact on phasic arousal.

## Experiment 3

The discrepancy in neutral picture ratings between experiments 1–2 may suggest that an arousing context is required to reveal an impact of sound unpredictability on ratings of neutral images. To address whether this is due to arousal associated with the visual context, or whether it requires a negatively valenced context, we revisited this question using negative, neutral and positive IAPS stimuli in experiment 3.

### Method

This experiment was similar to the first, with an additional third condition of positive arousing images, and thus followed a 2 (sound type: predictable/unpredictable) × 3 (picture type: negative, neutral, positive) factorial design. Forty participants (20 male, 20 female; age: M = 21.9; SD = 3.8 years) watched the 16 least arousing neutral, most arousing aversive and most arousing positive images (defined analogous to experiment 1, and excluding explicit nude images) from the IAPS, for 1 s each, in randomised order, and in one single block. ISI was 4.4 s. Responses were the same as in experiment 1. Two datasets were excluded: one due to technical malfunction, one due to lack of compliance with the instruction to rate the images by a key press.

### Results & Discussion

Valence influenced ratings (F(2, 72) = 418.0; η^2^ = .967; p < .001), RT (F(2, 72) = 6.3; η^2^ = .149; p < .01) and SCR (F(2, 72) = 8.8; η^2^ = .197; p < .001). Compared to neutral images, positive images were more often rated as positive (Table 2), and associated with higher SCR. In contrast, negative stimuli were more often rated as negative, associated with shorter RT, and with higher SCR. However, sound sequence had no impact on ratings, RT, or phasic stimulus-associated SCR, and there was no interaction of sound sequence and valence.

The lack of a sound sequences effect or interaction with valence suggests that the combination of negative and neutral pictures in experiment 1, rather than a generally arousing context, was crucial to the impact of unpredictability.

## Experiment 4

Using a different picture context than in experiment 1, experiments 2–3 provided null results. Hence, we sought to confirm results from experiment 1, again using a context of negative and neutral images. At the same time, to facilitate interpretation of our results with respect to a previous experiment employing a dot probe task with faces [15], we used similar stimuli, namely face photographs with angry, fearful, and neutral emotional expression.

### Method

Similar to experiment 3, in this experiment we implemented a 2 (sound type: predictable/unpredictable) × 3 (picture type: neutral, anger, fear) factorial design. Forty-two participants (21 male, 21 female; age: M = 25.2; SD = 4.0 years) watched, in randomised order, photographs from 38 actors of the Karolinska Directed Emotional Faces set [29] with angry, fearful, and neutral expression. The faces were partially masked to remove hair and clothing, and shown in grey scale on a black background. The experiment was divided into three blocks and lasted about 15 minutes. Event timing and responses were the same as in experiment 1.

### Results & Discussion

Overall, angry faces were rated negative more often than fearful faces, while fearful faces were rated negative more often than neutral faces (main effect valence, Tables 2 and 4, Fig 1). All faces were more often rated negative in the unpredictable than in the predictable condition (main effect sound) with no interaction. Post-hoc t-tests (2-tailed, uncorrected) revealed that this was due to more negative rating of neutral (t(40) = 2.1; p < .05) and fearful pictures (t(40) = 2.0; p < .05) while the impact on angry pictures failed to reach significance (t(40) = 1.7; p = .09). The effect of sound sequence survived (all p < .05) correction for baseline arousal, state anxiety, and trait anxiety. Reaction times were longer for neutral than angry or fearful faces (main effect valence), and were shorter in the unpredictable than in the predictable condition. Baseline tonic sympathetic arousal (SF) and phasic sympathetic arousal were not influenced by the sounds.

**Table 4:** Experiment 4: Results from a 2 (sound sequence) × 3 (facial expression) repeated-measures ANOVA on stimulus ratings, reaction times (RT), and phasic sympathetic arousal as estimated from skin conductance responses (SCR); and from a t-test on tonic sympathetic arousal as estimated from spontaneous fluctuations (SF) in skin conductance.

Rating	Expression	F (2, 80) = 57.5	η^2^ = .590	p < .001
	Sound	F (1,40) = 9.9	η^2^ = .198	p < .005
	Expression x Sound	F (2,80) < 1	η^2^ = .007	n. s.
RT	Expression	F (2, 80) = 14.7	η^2^ = .269	p < .001
	Sound	F (1,40) = 4.8	η^2^ = .108	p < .05
	Valence x Sound	F (2,80) < 1	η^2^ = .025	n. s.
SCR	Expression	F (2, 80) = 6.7	η^2^ = .144	p < .005
	Sound	F (1,40) < 1	η^2^ = .010	n. s.
	Expression x Sound	F (2,80) < 1	η^2^ = .008	n.s.
SF	Sound	t (40) = 1.2		n.s.

In summary, results from this experiment suggest that unpredictable sounds indeed alter the evaluation of neutral stimuli, but only when presented in a context of negative visual stimuli.

## Comparison of Experiments 1–4

So far, we have reported different effects on ratings of neutral images in experiments 1–4 without a direct statistical comparison. To formally test these differences, we combined responses to neutral stimuli which were presented in all four experiments. We analysed data alone from individuals who received predictable or unpredictable sound stimulation (i. e. excluding the silence condition which was not used in all four experiments), and initially confined our analysis to experiments 1–3 as these involved the same type of neutral (IAPS) pictures.

For picture ratings, this analysis revealed a significant interaction of the experiment and sound factors (F(2, 112) = 3.7; η^2^ = .061; p < .05), and a main effect of experiment (F(2, 112) = 48.7; η^2^ = .465; p < .001). This confirms differences between experiments concerning the impact of sound sequence on ratings of neutral images. In particular, the impact of unpredictable sounds on neutral picture ratings in experiment 1 was larger than in experiment 2 (post hoc contrast, p < .01) or experiment 3 (p = .09). Also, neutral pictures in experiment 2 were rated more negative than in experiment 1 (p < .001) or experiment 3 (p < .001).

There was no effect of sound or experiment on RTs. SCRs to neutral stimuli were significantly higher in the unpredictable than predictable condition (main effect sound, F(1, 112) = 4.3; η^2^ = .037;p < .05), and also differed between experiments (F(2, 112) = 4.6; η^2^ = .075; p < .05). SCR were similar between experiment 1 and 2 (p = .47) but smaller in experiment 3 compared to experiment 2 (p < .005).

All these effects were replicated when including neutral faces from experiment 4 together with the neutral IAPS pictures from experiments 1–3, again confirming the difference between experiments with respect to the effect of sound, and the overall phasic SCR increase in the unpredictable condition.

In summary, this analysis corroborated a significant impact of visual context on the impact of the unpredictable sound sequence. As a side finding, ratings of neutral pictures were also impacted by visual context; in particular they were rated more negative when presented on their own. This may be due to the fact that in the other two experiments, the contrast with the aversive images may have influenced their ratings. It will be interesting to investigate whether this would also occur if pictures were chosen from a continuum between neutral and negative.

## Experiment 5

Given an evaluation bias of visual stimuli in the context of the unpredictable sound sequence in experiment 1 and 4, we investigated how subjects evaluated the sound sequences themselves, both in terms of "wanting" and "liking" [30].

### Method

During 5 minutes, 72 participants (36 male, 36 female; age: M = 23.9; SD = 4.6 years) heard predictable or unpredictable sound sequences over headphones and could change between the two sequences ad libitum with a mouse click. The starting sequence was counterbalanced. After the experiment, participants were asked to rate which sequence they found more unpleasant, interesting, and activating, on a scale with 5 levels, anchored with *regular* and *irregular* as descriptions of the two sequences.

### Results & Discussion

In terms of "wanting", we analysed time spent with the sounds. Under the null hypothesis that there is no difference between the sequences, each participant would on average spent half the time with the unpredictable sequence, and half of the participants would spend more time with the unpredictable than with the predictable sequence. We found that the average time spent with the sounds did not differ from a uniform distribution (H_0_: 150 s; M (unpredictable) = 153.2 s; SEM = 9.9 s; t(71) = 0.32; p = .74), but that more than half of the sample spent more time with the unpredictable compared to predictable sound sequence (45/72; p < .05; binomial test). In terms of "liking", subjective ratings for each dimension ranged from -2 (predictable) to 2 (unpredictable), and were tested against zero. The sequences were rated as similarly unpleasant (0.15 ± 0.17; t(71) = 0.88; p = .38), and the unpredictable sound sequence was rated as more interesting (1.07 ± 0.11; t(71) = 9.37; p < .001) and more activating (0.48 ± 0.15; t(71) = 3.17; p < .01). There was no influence of state or trait anxiety on preference, or on the time spent with the regular sequence. Participants switched on the average 18.9 (SD = 2.1) times between the sequences; the number of switches was not related to any of the other dependent measures, or to state/trait anxiety levels.

To summarise, participants were more likely to spend longer with the unpredictable sound sequence. Thus, our results provide significant evidence against a suggestion that unpredictable sound sequence avoided. At the same time, there was no evidence that at a subjective level either sequence was perceived as more aversive than the other. The unpredictable sequence was rated as more interesting and activating.

## Experiment 6

Finally, we sought to further investigate anxiogenic properties of an unpredictable sound sequence. Anxiety can be indexed by subjective feeling or by tonic sympathetic arousal. Experiments 1, 2 and 4 did not provide evidence for an effect of sound sequence on tonic sympathetic arousal in the absence of stimulus presentation. Here, we replicated this in a within-subjects design where we increased sensitivity for detecting arousal changes by measuring heart rate. Further, because previous mouse experiments suggested an unpredictable sound sequence might specifically elicit anxiety, we investigated subjective feelings during presentation of the sound sequence to furnish qualitative differentiation of emotional states.

### Method

Experiment 6 followed a one-way factorial design with a within-subjects factor sound type (silence/predictable/unpredictable). Twenty-four participants (12 male, 12 female; age: M = 23.5; SD = 0.9 years) listened to 5 minutes of silence, predictable, and unpredictable sound sequences (2 minutes skin conductance and ECG recordings, 3 minutes self-report questionnaires) in counterbalanced order. As self-report measures, we used the multidimensional mood checklist (Befindlichkeitsskalierung anhand von Kategorien und Eigenschaftswörtern [BSKE], [31]), based upon the German Adjective Checklist (Eigenschaftswörterliste [EWL], [32]) with positively valenced subtests *relaxation*, *well-being*, *self-confidence*, *alertness*; and negatively valenced subtests *excitement*, *sulkiness*, *angriness*, *anxiety*, *depression*, *listlessness* with 2 items per subtest. Each item was described with one noun and two illustrating adjectives and rated on a scale from 0 (*not at all*) to 6 (*very strongly*). This instrument has been validated in the context of state anxiety [33, 34].

ECG was analysed by automatically counting R-spikes per each 2-minute epoch with visual control for artefacts (VisonAnalyser, BrainProducts). Heart rate quantified over short periods of time can be seen as a measure of combined autonomic input to heart [35] and has been validated in the context of experimental anxiety and mental stress [33, 34, 36]. Due to equipment malfunction, ECG was only available for 19 out of 24 participants.

### Results & Discussion

Even without correction for multiple comparisons, the unpredictable sound sequence had no effect on estimates of tonic sympathetic arousal (all *p* > .20). There were no subjective mood changes on negative dimensions induced by the unpredictable sound sequence (all *p* > .10). On positive dimensions, *relaxedness* was slightly reduced by the unpredictable sound sequence (3.25 ± 0.21 vs. 2.94 ± 0.30; F(1, 21) = 5.81; *p* < .05, not corrected for multiple comparison). The predictable (control) sound sequence induced a heart rate increase from the silence (baseline) condition (F(1, 17) = 7.21; *p* < .05) and an increase in feelings of *anger/aggression* (F(1, 21) = 7.16; *p* < .05) while having no effect on other measures. Even at the trend-level, there was no difference on these particular measures between the predictable and unpredictable sequence.

## General Discussion

In this paper, we revisit the questions of whether unpredictable, non-salient events change evaluation of unrelated stimuli, if they are by themselves evaluated as aversive, and if they are anxiogenic. First, we demonstrate that evaluation of neutral IAPS pictures and of neutral, angry and fearful face photographs is biased towards a higher percentage of negative ratings while participants are exposed to the unpredictable sound sequence. However, this evaluation bias only occurs when the presented picture set consists of neutral and negative pictures, but not when neutral and negative pictures are interspersed with positive pictures, or when neutral pictures are presented on their own. Specifically, we observed a significant interaction of the visual context with the sound sequences, on ratings of neutral pictures. Secondly, there is some evidence that unpredictable sound sequences, when presented on their own, are actively preferred in a forced-choice task–strongly suggesting that they are not aversive. Finally, there is no impact of the unpredictable sound sequence on negative mood dimensions, while a slight decrease in positive mood does not survive correction for multiple comparison. Tonic (baseline) sympathetic arousal is unaltered by the sound sequence in all experiments. Altogether, this suggests that anxiogenic properties of the sound sequences in humans are negligible when they are presented on their own.

The fact that evaluation of pictures is only changed when neutral and negative stimuli are presented in the same set (but not when combined with positive pictures or when only neutral pictures are presented) suggests that picture context is crucial for the expression of an impact of sound predictability. This finding lends itself to a number of interpretations. First, it is possible that the unpredictable sound sequence increases uncertainty in the evaluation of the pictures. Prior expectations, shaped by the visual context, might then exert a stronger influence on ratings, biasing them towards more negative in a context with negative pictures. We note that ratings of the different picture categories do not simply become more similar to each other. Although such a hypothesis might be construed from the cross-over interaction of picture type and sound sequence in experiment 1, all pictures including the (mildly) negative ones were rated more negative in experiment 4. As a second explanation for our finding, hearing an unpredictable sound sequence might increase arousal, and this could be misattributed to the pictures. This explanation mainly fits the data for neutral pictures. In a context of negative images, the possibility of having seen a negatively valenced picture constitutes a likely source of arousal. In a state of increased phasic arousal, a neutral picture might then be perceived and rated as negatively valenced. This should not occur in a context of positive and negative, or neutral images alone. The findings with respect to neutral images are in line with this interpretation, and phasic arousal is indeed increased in response to neutral pictures by the presence of unpredictable vs. predictable sounds across experiments 1 to 4. However, this explanation appears to be restricted to neutral images and therefore remains speculative. A possible way of testing this hypothesis would be to present only neutral and highly arousing positive pictures. According to the arousal hypotheses, ratings of the neutral pictures should then be rendered more positive. As a note of caution, these interpretations rely on a post-hoc analysis of responses to neutral picture across the different experiments. It would be desirable to compare the impact of image context on evaluation of neutral images within one experimental design.

We found that an unpredictable sound sequence is not evaluated as more aversive by itself. In fact, subjects actively preferred this sound sequence over a predictable control sequence, showing increased "wanting". Explicit evaluation of the two sound sequences in "liking" ratings indicated no difference in aversiveness, although the unpredictable sequences were rated as more interesting and activating. Finally, we found no evidence of anxiogenic properties for any of the sequences in subjective feeling. As a caveat, it is well known that when engaged in a task, unrelated unpredictable events are more distracting than predictable ones [37]. It is possible that an unpredictable, potentially more distracting, sequence would be evaluated more negative when engaged in a task as in experiments 1–4, but not when presented on its own.

Altered appraisal of context events can explain our previous findings about the effects of unpredictable neutral sounds in humans [15]. An increased attentional bias towards threatening information as found in this previous experiment might be induced by more negative appraisal of the stimuli used. Effects on rodent behaviour can also be explained from this perspective though we hasten to add we have not experimentally tested this possibility in rodents. In fact there might be species differences in the evaluation of unpredictability, or uncertainty, such that rodents raised in laboratories might be adapted to a much more predictable environment than humans, who are prone to perceive the absence of change as boring and monotonous [8, 9].

A final limitation that we need to acknowledge is that the rating scale used to assess picture evaluation was simple. This choice of a binary rating scale was motivated by the need to minimise possible distracting influences of sounds on the rating process. A more elaborate rating procedure might be harnessed to reveal more subtle effects particularly in the evaluation of highly arousing pictures.

In conclusion, we demonstrate that a sequence of unpredictable neutral events alters the evaluation of unrelated neutral pictures, and face photographs, but only when presented in a negatively valenced visual context. At the same time, this unpredictable sequence had no effect on tonic sympathetic arousal, negative subjective feeling, or subjective preference, when presented alone. Instead, participants were more likely to spend more time with the unpredictable than with the predictable sequence. This suggests that unpredictable sequence is neither anxiogenic nor aversive. This opens a new perspective on unpredictability and might help understanding human behaviour in ever-changing natural environments.
